# An indirect comparison of efficacy and safety of elvitegravir/cobicistat/emtricitabine/tenofovir and dolutegravir + abacavir/lamivudine

**DOI:** 10.7448/IAS.17.4.19779

**Published:** 2014-11-02

**Authors:** Felipe Rogatto, Stephane Bouee, Vivianne Jeanbat, David Piontkowsky, Filipa Aragao, Matthew Bosse

**Affiliations:** 1 Medical Affairs, Gilead Sciences Europe, Uxbridge, UK; 2 Pharmacoepidemiology, CEMKA, Bourg La Reine, France; 3 Medical Affairs, Gilead Sciences, Foster City, CA, USA

## Abstract

**Background:**

Integrase strand transfer inhibitors (INSTI) are the standard of care for naïve HIV-infected individuals due to their favourable efficacy and safety profile. The newest INSTIs, elvitegravir and dolutegravir, have not been evaluated in a head to head study; however, both have been compared to efavirenz/emtricitabine/tenofovir (EFV/FTC/TDF) in phase III trials. Elvitegravir/cobicistat/emtricitabine/tenofovir DF (E/C/F/TDF) was compared to EFV/FTC/TDF for 144 weeks in Gilead Study 102 (GS-102), while dolutegravir (DTG) with the abacavir/lamivudine fixed-dose combination (ABC/3TC) was compared to EFV/FTC/TDF for 96 weeks in the SINGLE study. The objective of this analysis is to perform an indirect comparison at 48 and 96 weeks of E/C/F/TDF to DTG+ABC/3TC by using the two trials evaluating each of these regimens compared to EFV/FTC/TDF.

**Methods:**

An indirect comparison was performed by using Bucher's methodology to calculate risk differences based on the two phase III clinical trials described above.

**Results:**

At week 48 (snapshot analysis), 88% of the patients on E/C/F/TDF and DTG+ABC/3TC had HIV RNA <50 c/mL, while 84% and 81% of patients on EFV/FTC/TDF were suppressed in GS-102 and SINGLE, respectively. At week 96, 84% of patients receiving E/C/F/TDF compared with 80% of patients receiving DTG+ABC/3TC remained suppressed, while 82% and 72% on EFV/FTC/TDF maintained HIV RNA <50 c/mL in GS-102 and SINGLE. At week 144 80% of patients on E/C/F/TDF remained suppressed (vs. 75% of the patients on EFV/FTC/TDF). Results of indirect comparison showed a risk difference of HIV RNA <50 copies per mL between E/C/F/TDF compared with DTG+ABC/3TC of −4% (CI 95%=−11 to 3) for the ITT 48 weeks (p=0.3) and −5% (95% CI=−13 to 3) for the ITT 96 weeks (p=0.2). In regards to safety, there was no significant difference between E/C/F/TDF and DTG+ABC/3TC for any adverse event (AE) (p=0.3), serious AEs (0.13), drug related AEs (0.7), or drug-related serious AEs (0.6).

**Conclusions:**

In GS-102 and SINGLE, 88% of the patients on E/C/F/TDF and DTG+ABC/3TC were virologically suppressed at week 48. At week 96, these proportions were 84% for E/C/F/TDF and 80% for DTG+ABC/3TC. The indirect efficacy comparisons between EVG/COBI/FTC/TDF and DTG+ABC/3TC at week 48 and 96 revealed no statistically significant differences.

